# Supplementary material for: Penalized Reduced Rank Regression for Multi‐Outcome Survival Data Supports a Common Metabolic Risk Score for Age‐Related Diseases
Source: Stat Med. 2025 Jul 15;44(15-17):e70156. doi: 10.1002/sim.70156 (PMC12261392; doi:10.1002/sim.70156)
Supplement: Supplementary file 1 — Data S1. Supporting Information S1. [file SIM-44-0-s001.pdf]

Supporting Information to "Penalized reduced rank regression for multi-outcome survival data supports a common metabolic risk score for age-related diseases"

Marije H. Sluiskes[1], Hein Putter[1], Marian Beekman[1], Jelle J. Goeman[1] and Mar Rodríguez-Girondo[1]

[1]Biomedical Data Sciences, Leiden University Medical Center, Einthovenweg 20, 2333 ZC Leiden, The Netherlands

Appendix A

The calculation of the MetaboHealth score as described below follows the procedure outlined in the original publication by Deelen et al. (2019):

1. Use the same training and test data sets as defined when fitting the penalized survRRR model. As we do not refit the MetaboHealth model, we only consider the test data set.
2. In the test data set, start from the original (raw) metabolic variable values. (For MetaboHealth, the metabolic variables need to be log-transformed before centering and scaling. When fitting the penalized survRRR model, metabolic variables were centered and scaled without a log-transformation.)
3. Add a value of 1 to the raw metabolic variables, log-transform, center and scale.
4. Calculate the MetaboHealth score as: $\mathit{MetaboHealth} = \log(0.8) \times \mathit{xxl\_vldl\_l} + \log(0.87) \times \mathit{s\_hdl\_l} + \log(0.85) \times \mathit{vldl\_size} + \log(0.78) \times \mathit{pufa\_pct} + \log(1.16) \times \mathit{glucose} + \log(1.06) \times \mathit{lactate} + \log(0.93) \times \mathit{his} + \log(1.23) \times \mathit{ile} + \log(0.82) \times \mathit{leu} + \log(0.87) \times \mathit{val} + \log(1.13) \times \mathit{phe} + \log(1.08) \times \mathit{acetoacetate} + \log(0.89) \times \mathit{albumin} + \log(1.32) \times \mathit{glyca}$. Here, the metabolite names already refer to the transformed metabolites as defined under step 3.

References

Deelen, J., Kettunen, J., Fischer, K., van Der Spek, A., Trompet, S., Kastenmüller, G., Boyd, A., Zierer, J., van Den Akker, E. B., Ala-Korpela, M., Amin, N., Demirkan, A., Ghanbari, M., van Heemst, D., Ikram, M. A., van Klinken, J. B., Mooijaart, S. P., Peters, A., Salomaa, V., Sattar, N., Spector, T. D., Tiemeier, H., Verhoeven, A., Waldenberger, M., Würtz, P., Smith, G. D., Metspalu, A., Perola, M., Menni, C., Geleijnse, J. M., Drenos, F., Beekman, M., Jukema, J. W., van Duijn, C. M., and Slagboom, P. E. (2019). A metabolic profile of all-cause mortality risk identified in an observational study of 44,168 individuals. *Nature Communications*, 10(1):3346.
