# Supplementary material for: Penalized Reduced Rank Regression for Multi‐Outcome Survival Data Supports a Common Metabolic Risk Score for Age‐Related Diseases
Source: Stat Med. 2025 Jul 15;44(15-17):e70156. doi: 10.1002/sim.70156 (PMC12261392; doi:10.1002/sim.70156)
Supplement: Supplementary file 2 — Data S2. Supporting Information S2. [file SIM-44-0-s008.pdf]

Supporting Information to “Penalized reduced rank regression for multi-outcome survival data supports a common metabolic risk score for age-related diseases”

Marije H. Sluiskes[1], Hein Putter[1], Marian Beekman[1],
Jelle J. Goeman[1] and Mar Rodríguez-Girondo[1]

[1]Biomedical Data Sciences, Leiden University Medical Center, Einthovenweg 20, 2333 ZC Leiden, The Netherlands

Appendix B

A biplot (Gabriel, 1971) graphically displays the results of a rank 2 model. It can be used to plot the effects of matrices **A** and **Γ** jointly (ter Braak and Looman, 1994; Fiocco et al., 2006).

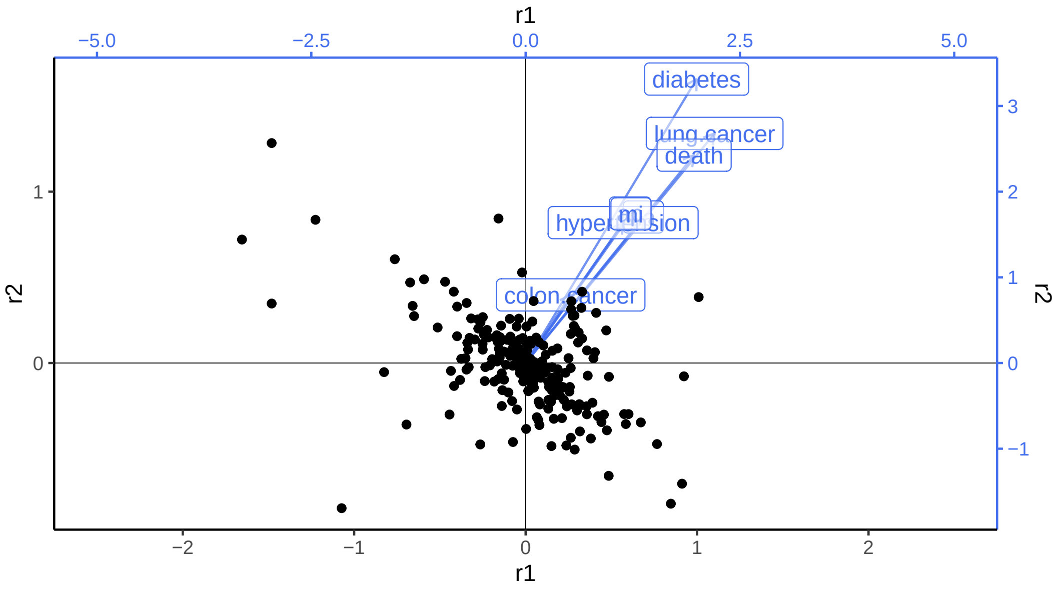

Figure 1: Biplot of the optimal rank 2 model. The blue-colored axes correspond to the position of the blue arrows; the black-colored axes correspond to the position of the black dots.

Figure 1 shows the biplot of the optimal rank 2 model, of the model as described in the main manuscript. The effect of **Γ**, i.e. effect of the rank scores on the age-related outcomes are represented by arrows (in blue). The effect of **A**, i.e. the effect of the metabolic variables on the rank scores are represented by dots (in black). Vectors pointing in the same direction suggest a positive correlation; opposite directions suggest a negative correlation and a 90-degree angle indicates no correlation. The

impact of each predictor on the outcome can be evaluated by projecting each black dot on the vectors of the outcomes.

The biplot shows that all aging-related outcomes point in the same direction. This suggests a rank 1 model. The outcomes are not represented by a single rank, but by a combination: they form approximately a 45 degree angle with the axes of both ranks. The projection of the metabolites on the vectors of the outcomes is generally not that large, indicating that their predictive strength is not strong. Metabolites with a strong effect on one rank tend to have a strong opposite effect on the other rank, such that their total effect on any given outcome is small.

References

Fiocco, M., Putter, H., Van de Velde, C., and Van Houwelingen, J. (2006). Reduced rank proportional hazards model for competing risks: an application to a breast cancer trial. *Journal of Statistical Planning and Inference*, 136(5):1655–1668.

Gabriel, K. R. (1971). The biplot graphic display of matrices with application to principal component analysis. *Biometrika*, 58(3):453–467.

ter Braak, C. J. and Looman, C. W. (1994). Biplots in reduced-rank regression. *Biometrical Journal*, 36(8):983–1003.
