# Supplementary material for: Penalized Reduced Rank Regression for Multi‐Outcome Survival Data Supports a Common Metabolic Risk Score for Age‐Related Diseases
Source: Stat Med. 2025 Jul 15;44(15-17):e70156. doi: 10.1002/sim.70156 (PMC12261392; doi:10.1002/sim.70156)
Supplement: Supplementary file 3 — Data S3. Supporting Information S3. [file SIM-44-0-s005.pdf]

Supporting Information to "Penalized reduced rank regression for multi-outcome survival data supports a common metabolic risk score for age-related diseases"

Marije H. Sluiskes[1], Hein Putter[1], Marian Beekman[1],
Jelle J. Goeman[1] and Mar Rodríguez-Girondo[1]

[1]Biomedical Data Sciences, Leiden University Medical Center,
Einthovenweg 20, 2333 ZC Leiden, The Netherlands

SUPPORTING TABLE 1 Overview of all included metabolic measures from Nightingale Health Ltd., including unit of measurement and corresponding UKB Field ID. Metabolic variables in shaded rows (7 out of 249) were excluded due to not meeting the selection criteria.

Metabolic variable	Description	Unit of measurement	UKB Field ID
acetate	acetate	mmol/L	23475
acetoacetate	acetoacetate	mmol/L	23476
acetone	acetone	mmol/L	23477
ala	alanine	mmol/L	23460
albumin	albumin	g/l	23479
apoa1	apolipoprotein a1	g/l	23440
apob	apolipoprotein b	g/l	23439
apob_by_apoa1	apolipoprotein b to apolipoprotein a1 ratio	ratio	23441
bohbutyrate	3-hydroxybutyrate	mmol/L	23474
cholines	total cholines	mmol/L	23436
citrate	citrate	mmol/L	23473
clinical_ldl_c	clinical ldl cholesterol	mmol/L	23404
creatinine	creatinine	mmol/L	23478
dha	docosahexaenoic acid	mmol/L	23450
dha_pct	docosahexaenoic acid to total fatty acids percentage	%	23457
gln	glutamine	mmol/L	23461
glucose	glucose	mmol/L	23470
gly	glycine	mmol/L	23462
glyca	glycoprotein acetyls	mmol/L	23480
hdl_c	hdl cholesterol	mmol/L	23406
hdl_ce	cholesteryl esters in hdl	mmol/L	23418
hdl_fc	free cholesterol in hdl	mmol/L	23422
hdl_l	total lipids in hdl	mmol/L	23426
hdl_p	concentration of hdl particles	mmol/L	23430
hdl_pl	phospholipids in hdl	mmol/L	23414
hdl_size	average diameter for hdl particles	nm	23433
hdl_tg	triglycerides in hdl	mmol/L	23410
his	histidine	mmol/L	23463

idl_c	cholesterol in idl	mmol/L	23526
idl_c_pct	cholesterol to total lipids in idl percentage	%	23610
idl_ce	cholesteryl esters in idl	mmol/L	23527
idl_ce_pct	cholesteryl esters to total lipids in idl percentage	%	23611
idl_fc	free cholesterol in idl	mmol/L	23528
idl_fc_pct	free cholesterol to total lipids in idl percentage	%	23612
idl_l	total lipids in idl	mmol/L	23524
idl_p	concentration of idl particles	mmol/L	23523
idl_pl	phospholipids in idl	mmol/L	23525
idl_pl_pct	phospholipids to total lipids in idl percentage	%	23609
idl_tg	triglycerides in idl	mmol/L	23529
idl_tg_pct	triglycerides to total lipids in idl percentage	%	23613
ile	isoleucine	mmol/L	23465
l_hdl_c	cholesterol in large hdl	mmol/L	23561
l_hdl_c_pct	cholesterol to total lipids in large hdl percentage	%	23635
l_hdl_ce	cholesteryl esters in large hdl	mmol/L	23562
l_hdl_ce_pct	cholesteryl esters to total lipids in large hdl percentage	%	23636
l_hdl_fc	free cholesterol in large hdl	mmol/L	23563
l_hdl_fc_pct	free cholesterol to total lipids in large hdl percentage	%	23637
l_hdl_l	total lipids in large hdl	mmol/L	23559
l_hdl_p	concentration of large hdl particles	mmol/L	23558
l_hdl_pl	phospholipids in large hdl	mmol/L	23560
l_hdl_pl_pct	phospholipids to total lipids in large hdl percentage	%	23634
l_hdl_tg	triglycerides in large hdl	mmol/L	23564
l_hdl_tg_pct	triglycerides to total lipids in large hdl percentage	%	23638
l_ldl_c	cholesterol in large ldl	mmol/L	23533
l_ldl_c_pct	cholesterol to total lipids in large ldl percentage	%	23615
l_ldl_ce	cholesteryl esters in large ldl	mmol/L	23534
l_ldl_ce_pct	cholesteryl esters to total lipids in large ldl percentage	%	23616
l_ldl_fc	free cholesterol in large ldl	mmol/L	23535
l_ldl_fc_pct	free cholesterol to total lipids in large ldl percentage	%	23617
l_ldl_l	total lipids in large ldl	mmol/L	23531
l_ldl_p	concentration of large ldl particles	mmol/L	23530
l_ldl_pl	phospholipids in large ldl	mmol/L	23532
l_ldl_pl_pct	phospholipids to total lipids in large ldl percentage	%	23614
l_ldl_tg	triglycerides in large ldl	mmol/L	23536
l_ldl_tg_pct	triglycerides to total lipids in large ldl percentage	%	23618

l_vldl_c	cholesterol in large vldl	mmol/L	23498
l_vldl_c_pct	cholesterol to total lipids in large vldl percentage	%	23590
l_vldl_ce	cholesteryl esters in large vldl	mmol/L	23499
l_vldl_ce_pct	cholesteryl esters to total lipids in large vldl percentage	%	23591
l_vldl_fc	free cholesterol in large vldl	mmol/L	23500
l_vldl_fc_pct	free cholesterol to total lipids in large vldl percentage	%	23592
l_vldl_l	total lipids in large vldl	mmol/L	23496
l_vldl_p	concentration of large vldl particles	mmol/L	23495
l_vldl_pl	phospholipids in large vldl	mmol/L	23497
l_vldl_pl_pct	phospholipids to total lipids in large vldl percentage	%	23589
l_vldl_tg	triglycerides in large vldl	mmol/L	23501
l_vldl_tg_pct	triglycerides to total lipids in large vldl percentage	%	23593
la	linoleic acid	mmol/L	23449
la_pct	linoleic acid to total fatty acids percentage	%	23456
lactate	lactate	mmol/L	23471
ldl_c	ldl cholesterol	mmol/L	23405
ldl_ce	cholesteryl esters in ldl	mmol/L	23417
ldl_fc	free cholesterol in ldl	mmol/L	23421
ldl_l	total lipids in ldl	mmol/L	23425
ldl_p	concentration of ldl particles	mmol/L	23429
ldl_pl	phospholipids in ldl	mmol/L	23413
ldl_size	average diameter for ldl particles	nm	23432
ldl_tg	triglycerides in ldl	mmol/L	23409
leu	leucine	mmol/L	23466
m_hdl_c	cholesterol in medium hdl	mmol/L	23568
m_hdl_c_pct	cholesterol to total lipids in medium hdl percentage	%	23640
m_hdl_ce	cholesteryl esters in medium hdl	mmol/L	23569
m_hdl_ce_pct	cholesteryl esters to total lipids in medium hdl percentage	%	23641
m_hdl_fc	free cholesterol in medium hdl	mmol/L	23570
m_hdl_fc_pct	free cholesterol to total lipids in medium hdl percentage	%	23642
m_hdl_l	total lipids in medium hdl	mmol/L	23566
m_hdl_p	concentration of medium hdl particles	mmol/L	23565
m_hdl_pl	phospholipids in medium hdl	mmol/L	23567
m_hdl_pl_pct	phospholipids to total lipids in medium hdl percentage	%	23639
m_hdl_tg	triglycerides in medium hdl	mmol/L	23571
m_hdl_tg_pct	triglycerides to total lipids in medium hdl percentage	%	23643
m_ldl_c	cholesterol in medium ldl	mmol/L	23540
m_ldl_c_pct	cholesterol to total lipids in medium ldl percentage	%	23620

m_ldl_ce	cholesteryl esters in medium ldl	mmol/L	23541
m_ldl_ce_pct	cholesteryl esters to total lipids in medium ldl percentage	%	23621
m_ldl_fc	free cholesterol in medium ldl	mmol/L	23542
m_ldl_fc_pct	free cholesterol to total lipids in medium ldl percentage	%	23622
m_ldl_l	total lipids in medium ldl	mmol/L	23538
m_ldl_p	concentration of medium ldl particles	mmol/L	23537
m_ldl_pl	phospholipids in medium ldl	mmol/L	23539
m_ldl_pl_pct	phospholipids to total lipids in medium ldl percentage	%	23619
m_ldl_tg	triglycerides in medium ldl	mmol/L	23543
m_ldl_tg_pct	triglycerides to total lipids in medium ldl percentage	%	23623
m_vldl_c	cholesterol in medium vldl	mmol/L	23505
m_vldl_c_pct	cholesterol to total lipids in medium vldl percentage	%	23595
m_vldl_ce	cholesteryl esters in medium vldl	mmol/L	23506
m_vldl_ce_pct	cholesteryl esters to total lipids in medium vldl percentage	%	23596
m_vldl_fc	free cholesterol in medium vldl	mmol/L	23507
m_vldl_fc_pct	free cholesterol to total lipids in medium vldl percentage	%	23597
m_vldl_l	total lipids in medium vldl	mmol/L	23503
m_vldl_p	concentration of medium vldl particles	mmol/L	23502
m_vldl_pl	phospholipids in medium vldl	mmol/L	23504
m_vldl_pl_pct	phospholipids to total lipids in medium vldl percentage	%	23594
m_vldl_tg	triglycerides in medium vldl	mmol/L	23508
m_vldl_tg_pct	triglycerides to total lipids in medium vldl percentage	%	23598
mufa	monounsaturated fatty acids	mmol/L	23447
mufa_pct	monounsaturated fatty acids to total fatty acids percentage	%	23454
non_hdl_c	total cholesterol minus hdl-c	mmol/L	23401
omega_3	omega-3 fatty acids	mmol/L	23444
omega_3_pct	omega-3 fatty acids to total fatty acids percentage	%	23451
omega_6	omega-6 fatty acids	mmol/L	23445
omega_6_by_omega_3	omega-6 fatty acids to omega-3 fatty acids ratio	ratio	23459
omega_6_pct	omega-6 fatty acids to total fatty acids percentage	%	23452
phe	phenylalanine	mmol/L	23468
phosphatidylc	phosphatidylcholines	mmol/L	23437
phosphoglyc	phosphoglycerides	mmol/L	23434
pufa	polyunsaturated fatty acids	mmol/L	23446
pufa_by_mufa	polyunsaturated fatty acids to monounsaturated fatty acids ratio	ratio	23458

pufa_pct	polyunsaturated fatty acids to total fatty acids percentage	%	23453
pyruvate	pyruvate	mmol/L	23472
remnant_c	remnant cholesterol (non-hdl, non-ldl -cholesterol)	mmol/L	23402
s_hdl_c	cholesterol in small hdl	mmol/L	23575
s_hdl_c_pct	cholesterol to total lipids in small hdl percentage	%	23645
s_hdl_ce	cholesteryl esters in small hdl	mmol/L	23576
s_hdl_ce_pct	cholesteryl esters to total lipids in small hdl percentage	%	23646
s_hdl_fc	free cholesterol in small hdl	mmol/L	23577
s_hdl_fc_pct	free cholesterol to total lipids in small hdl percentage	%	23647
s_hdl_l	total lipids in small hdl	mmol/L	23573
s_hdl_p	concentration of small hdl particles	mmol/L	23572
s_hdl_pl	phospholipids in small hdl	mmol/L	23574
s_hdl_pl_pct	phospholipids to total lipids in small hdl percentage	%	23644
s_hdl_tg	triglycerides in small hdl	mmol/L	23578
s_hdl_tg_pct	triglycerides to total lipids in small hdl percentage	%	23648
s_ldl_c	cholesterol in small ldl	mmol/L	23547
s_ldl_c_pct	cholesterol to total lipids in small ldl percentage	%	23625
s_ldl_ce	cholesteryl esters in small ldl	mmol/L	23548
s_ldl_ce_pct	cholesteryl esters to total lipids in small ldl percentage	%	23626
s_ldl_fc	free cholesterol in small ldl	mmol/L	23549
s_ldl_fc_pct	free cholesterol to total lipids in small ldl percentage	%	23627
s_ldl_l	total lipids in small ldl	mmol/L	23545
s_ldl_p	concentration of small ldl particles	mmol/L	23544
s_ldl_pl	phospholipids in small ldl	mmol/L	23546
s_ldl_pl_pct	phospholipids to total lipids in small ldl percentage	%	23624
s_ldl_tg	triglycerides in small ldl	mmol/L	23550
s_ldl_tg_pct	triglycerides to total lipids in small ldl percentage	%	23628
s_vldl_c	cholesterol in small vldl	mmol/L	23512
s_vldl_c_pct	cholesterol to total lipids in small vldl percentage	%	23600
s_vldl_ce	cholesteryl esters in small vldl	mmol/L	23513
s_vldl_ce_pct	cholesteryl esters to total lipids in small vldl percentage	%	23601
s_vldl_fc	free cholesterol in small vldl	mmol/L	23514
s_vldl_fc_pct	free cholesterol to total lipids in small vldl percentage	%	23602
s_vldl_l	total lipids in small vldl	mmol/L	23510
s_vldl_p	concentration of small vldl particles	mmol/L	23509

s_vldl_pl	phospholipids in small vldl	mmol/L	23511
s_vldl_pl_pct	phospholipids to total lipids in small vldl percentage	%	23599
s_vldl_tg	triglycerides in small vldl	mmol/L	23515
s_vldl_tg_pct	triglycerides to total lipids in small vldl percentage	%	23603
sfa	saturated fatty acids	mmol/L	23448
sfa_pct	saturated fatty acids to total fatty acids percentage	%	23455
sphingomyelins	sphingomyelins	mmol/L	23438
tg_by_pg	triglycerides to phosphoglycerides ratio	ratio	23435
total_bcaa	total concentration of branched-chain amino acids (leucine + isoleucine + valine)	mmol/L	23464
total_c	total cholesterol	mmol/L	23400
total_ce	total esterified cholesterol	mmol/L	23415
total_fa	total fatty acids	mmol/L	23442
total_fc	total free cholesterol	mmol/L	23419
total_l	total lipids in lipoprotein particles	mmol/L	23423
total_p	total concentration of lipoprotein particles	mmol/L	23427
total_pl	total phospholipids in lipoprotein particles	mmol/L	23411
total_tg	total triglycerides	mmol/L	23407
tyr	tyrosine	mmol/L	23469
unsaturation	degree of unsaturation	degree	23443
val	valine	mmol/L	23467
vldl_c	vldl cholesterol	mmol/L	23403
vldl_ce	cholesteryl esters in vldl	mmol/L	23416
vldl_fc	free cholesterol in vldl	mmol/L	23420
vldl_l	total lipids in vldl	mmol/L	23424
vldl_p	concentration of vldl particles	mmol/L	23428
vldl_pl	phospholipids in vldl	mmol/L	23412
vldl_size	average diameter for vldl particles	nm	23431
vldl_tg	triglycerides in vldl	mmol/L	23408
xl_hdl_c	cholesterol in very large hdl	mmol/L	23554
xl_hdl_c_pct	cholesterol to total lipids in very large hdl percentage	%	23630
xl_hdl_ce	cholesteryl esters in very large hdl	mmol/L	23555
xl_hdl_ce_pct	cholesteryl esters to total lipids in very large hdl percentage	%	23631
xl_hdl_fc	free cholesterol in very large hdl	mmol/L	23556
xl_hdl_fc_pct	free cholesterol to total lipids in very large hdl percentage	%	23632
xl_hdl_l	total lipids in very large hdl	mmol/L	23552
xl_hdl_p	concentration of very large hdl particles	mmol/L	23551
xl_hdl_pl	phospholipids in very large hdl	mmol/L	23553
xl_hdl_pl_pct	phospholipids to total lipids in very large hdl percentage	%	23629
xl_hdl_tg	triglycerides in very large hdl	mmol/L	23557
xl_hdl_tg_pct	triglycerides to total lipids in very large hdl percentage	%	23633

xl_vldl_c	cholesterol in very large vldl	mmol/L	23491
xl_vldl_c_pct	cholesterol to total lipids in very large vldl percentage	%	23585
xl_vldl_ce	cholesteryl esters in very large vldl	mmol/L	23492
xl_vldl_ce_pct	cholesteryl esters to total lipids in very large vldl percentage	%	23586
xl_vldl_fc	free cholesterol in very large vldl	mmol/L	23493
xl_vldl_fc_pct	free cholesterol to total lipids in very large vldl percentage	%	23587
xl_vldl_l	total lipids in very large vldl	mmol/L	23489
xl_vldl_p	concentration of very large vldl particles	mmol/L	23488
xl_vldl_pl	phospholipids in very large vldl	mmol/L	23490
xl_vldl_pl_pct	phospholipids to total lipids in very large vldl percentage	%	23584
xl_vldl_tg	triglycerides in very large vldl	mmol/L	23494
xl_vldl_tg_pct	triglycerides to total lipids in very large vldl percentage	%	23588
xs_vldl_c	cholesterol in very small vldl	mmol/L	23519
xs_vldl_c_pct	cholesterol to total lipids in very small vldl percentage	%	23605
xs_vldl_ce	cholesteryl esters in very small vldl	mmol/L	23520
xs_vldl_ce_pct	cholesteryl esters to total lipids in very small vldl percentage	%	23606
xs_vldl_fc	free cholesterol in very small vldl	mmol/L	23521
xs_vldl_fc_pct	free cholesterol to total lipids in very small vldl percentage	%	23607
xs_vldl_l	total lipids in very small vldl	mmol/L	23517
xs_vldl_p	concentration of very small vldl particles	mmol/L	23516
xs_vldl_pl	phospholipids in very small vldl	mmol/L	23518
xs_vldl_pl_pct	phospholipids to total lipids in very small vldl percentage	%	23604
xs_vldl_tg	triglycerides in very small vldl	mmol/L	23522
xs_vldl_tg_pct	triglycerides to total lipids in very small vldl percentage	%	23608
xxl_vldl_c	cholesterol in chylomicrons and extremely large vldl	mmol/L	23484
xxl_vldl_c_pct	cholesterol to total lipids in chylomicrons and extremely large vldl percentage	%	23580
xxl_vldl_ce	cholesteryl esters in chylomicrons and extremely large vldl	mmol/L	23485
xxl_vldl_ce_pct	cholesteryl esters to total lipids in chylomicrons and extremely large vldl percentage	%	23581
xxl_vldl_fc	free cholesterol in chylomicrons and extremely large vldl	mmol/L	23486
xxl_vldl_fc_pct	free cholesterol to total lipids in chylomicrons and extremely large vldl percentage	%	23582
xxl_vldl_l	total lipids in chylomicrons and extremely large vldl	mmol/L	23482

xxl_vldl_p	concentration of chylomicrons and extremely large vldl particles	mmol/L	23481
xxl_vldl_pl	phospholipids in chylomicrons and extremely large vldl	mmol/L	23483
xxl_vldl_pl_pct	phospholipids to total lipids in chylomicrons and extremely large vldl percentage	%	23579
xxl_vldl_tg	triglycerides in chylomicrons and extremely large vldl	mmol/L	23487
xxl_vldl_tg_pct	triglycerides to total lipids in chylomicrons and extremely large vldl percentage	%	23583
