# Supplementary material for: Penalized Reduced Rank Regression for Multi‐Outcome Survival Data Supports a Common Metabolic Risk Score for Age‐Related Diseases
Source: Stat Med. 2025 Jul 15;44(15-17):e70156. doi: 10.1002/sim.70156 (PMC12261392; doi:10.1002/sim.70156)
Supplement: Supplementary file 4 — Data S4. Supporting Information S4. [file SIM-44-0-s007.pdf]

# Supporting Information to "Penalized reduced rank regression for multi-outcome survival data supports a common metabolic risk score for age-related diseases"

Marije H. Sluiskes[1], Hein Putter[1], Marian Beekman[1],
Jelle J. Goeman[1] and Mar Rodríguez-Girondo[1]

[1]Biomedical Data Sciences, Leiden University Medical Center,
Einthovenweg 20, 2333 ZC Leiden, The Netherlands

**SUPPORTING TABLE 2** Overview of the included diseases, corresponding ICD-10 codes and UKB Field IDs.

| Disease | ICD-10 | UKB Field ID | Description |
|---|---|---|---|
| Diabetes | E10 | 130706 | Date E10 first reported (insulin-dependent diabetes mellitus) |
| Diabetes | E11 | 130708 | Date E11 first reported (non-insulin-dependent diabetes mellitus) |
| Diabetes | E12 | 130710 | Date E12 first reported (malnutrition-related diabetes mellitus) |
| Diabetes | E13 | 130712 | Date E13 first reported (other specified diabetes mellitus) |
| Diabetes | E14 | 130714 | Date E14 first reported (unspecified diabetes mellitus) |
| AP | I20 | 131296 | Date I20 first reported (angina pectoris) |
| TIA | I60 | 131360 | Date I60 first reported (subarachnoid haemorrhage) |
| TIA | I61 | 131362 | Date I61 first reported (intracerebral haemorrhage) |
| TIA | I62 | 131364 | Date I62 first reported (other nontraumatic intracranial haemorrhage) |
| TIA | I63 | 131366 | Date I63 first reported (cerebral infarction) |
| TIA | I64 | 131368 | Date I64 first reported (stroke, not specified as haemorrhage or infarction) |
| TIA | I65 | 131370 | Date I65 first reported (occlusion and stenosis of precerebral arteries, not resulting in cerebral infarction) |
| TIA | I66 | 131372 | Date I66 first reported (occlusion and stenosis of cerebral arteries, not resulting in cerebral infarction) |
| TIA | I67 | 131374 | Date I67 first reported (other cerebrovascular diseases) |
| TIA | I68 | 131376 | Date I68 first reported (cerebrovascular disorders in diseases classified elsewhere) |
| TIA | I69 | 131378 | Date I69 first reported (sequelae of cerebrovascular disease) |
| MI | I21 | 131298 | Date I21 first reported (acute myocardial infarction) |
| Hpertension | I10 | 131286 | Date I10 first reported (essential (primary) hypertension) |

| | | | |
|---|---|---|---|
| Cancer | See below | 40006 | Type of cancer: ICD10 |
| Cancer | See below | 40005 | Date of cancer diagnosis |

**Cancer codes selected from UKB Field ID 40006:**

Lung: C340, C341, C342, C343, C348, C349

Colon: C180:C189, C19, C20
