# Supplementary material for: Penalized Reduced Rank Regression for Multi‐Outcome Survival Data Supports a Common Metabolic Risk Score for Age‐Related Diseases
Source: Stat Med. 2025 Jul 15;44(15-17):e70156. doi: 10.1002/sim.70156 (PMC12261392; doi:10.1002/sim.70156)
Supplement: Supplementary file 5 — Data S5. Supporting Information S5. [file SIM-44-0-s004.pdf]

Supporting Information to “Penalized reduced rank regression for multi-outcome survival data supports a common metabolic risk score for age-related diseases”

Marije H. Sluiskes[1], Hein Putter[1], Marian Beekman[1],
Jelle J. Goeman[1] and Mar Rodríguez-Girondo[1]

[1]Biomedical Data Sciences, Leiden University Medical Center,
Einthovenweg 20, 2333 ZC Leiden, The Netherlands

SUPPORTING TABLE 3 Number of events per outcome, before as well as after the age selection.

	Initial sample		Step 1: selecting those aged 50 and up		Step 2: selecting those at risk	
**Outcome**	**No**	**Yes**	**No**	**Yes**	**No**	**Yes**
Diabetes	92761	9888	69919	8634	69919	4231
TIA	97064	5585	73387	5166	73387	3627
Hypertension	62939	39710	43455	35098	43455	11763
AP	95076	7573	71522	7031	71522	3253
MI	97933	4716	74201	4352	74201	2173
Lung cancer	101676	973	77627	926	77627	864
Colon cancer	101413	1236	77404	1149	77404	876
Death	95020	7629	71421	7132	71421	7132
