# Supplementary material for: Penalized Reduced Rank Regression for Multi‐Outcome Survival Data Supports a Common Metabolic Risk Score for Age‐Related Diseases
Source: Stat Med. 2025 Jul 15;44(15-17):e70156. doi: 10.1002/sim.70156 (PMC12261392; doi:10.1002/sim.70156)
Supplement: Supplementary file 6 — Data S6. Supporting Information S6. [file SIM-44-0-s003.pdf]

Supporting Information to "Penalized reduced rank regression for multi-outcome survival data supports a common metabolic risk score for age-related diseases"

Marije H. Sluiskes[1], Hein Putter[1], Marian Beekman[1],
Jelle J. Goeman[1] and Mar Rodríguez-Girondo[1]

[1]Biomedical Data Sciences, Leiden University Medical Center,
Einthovenweg 20, 2333 ZC Leiden, The Netherlands

SUPPORTING TABLE 4 Initial conditions for the different runs, for both rank 1 (top) and rank 2 (bottom) models.

Rank 1				
**Run**	**Lambda grid**	**Initial Gamma**	**Seed**	**Convergence criterium**
1	lseq(from = 3e-4, to = 3e-6, length.out = 20)	matrix(rnorm(ranks*K),ranks,K)	500	5,00E-05
2	lseq(from = 3e-4, to = 3e-6, length.out = 20)	matrix(rnorm(ranks*K),ranks,K)	5	5,00E-05
3	lseq(from = 3e-4, to = 3e-6, length.out = 20)	matrix(rep(1, ranks*K),ranks,K)	500	5,00E-05
4	lseq(from = 4e-4, to = 3e-6, length.out = 15)	matrix(rep(1, ranks*K),ranks,K)	500	5,00E-05
5	lseq(from = 3e-4, to = 3e-6, length.out = 10)	matrix(runif(ranks*K),ranks,K)	500	5,00E-05

Rank 2				
**Run**	**Lambda grid**	**Initial Gamma**	**Seed**	**Convergence criterium**
1	lseq(from = 6e-4, to = 1e-8, length.out = 30)	matrix(rnorm(ranks*K),ranks,K)	5	5,00E-05
2	lseq(from = 6e-4, to = 1e-6, length.out = 20)	matrix(rnorm(ranks*K),ranks,K)	5	5,00E-05
3	lseq(from = 8e-4, to = 1e-8, length.out = 20)	matrix(runif(ranks*K),ranks,K)	500	5,00E-05
4	lseq(from = 6e-4, to = 1e-6, length.out = 20)	matrix(rnorm(ranks*K),ranks,K)	5	1,00E-06
5	lseq(from = 6e-4, to = 1e-9, length.out = 25)	matrix(rnorm(ranks*K),ranks,K)	5	1,00E-06
