# Supplementary material for: Penalized Reduced Rank Regression for Multi‐Outcome Survival Data Supports a Common Metabolic Risk Score for Age‐Related Diseases
Source: Stat Med. 2025 Jul 15;44(15-17):e70156. doi: 10.1002/sim.70156 (PMC12261392; doi:10.1002/sim.70156)
Supplement: Supplementary file 7 — Data S7. Supporting Information S7. [file SIM-44-0-s006.pdf]

Supporting Information to "Penalized reduced rank regression for multi-outcome survival data supports a common metabolic risk score for age-related diseases"

Marije H. Sluiskes[1], Hein Putter[1], Marian Beekman[1],
Jelle J. Goeman[1] and Mar Rodríguez-Girondo[1]

[1]Biomedical Data Sciences, Leiden University Medical Center,
Einthovenweg 20, 2333 ZC Leiden, The Netherlands

SUPPORTING TABLE 5 Estimated coefficient matrix $\hat{\boldsymbol{B}}$ of optimal model.

metabolic_variable	diabetes	tia	hypertension	ap	mi	lung_cancer	colon_cancer	death
total_c	0,923	0,268	0,378	0,394	0,372	0,387	0,194	0,388
non_hdl_c	0,145	0,042	0,059	0,062	0,058	0,061	0,03	0,061
remnant_c	0,629	0,183	0,258	0,269	0,254	0,264	0,132	0,264
vldl_c	0,512	0,149	0,21	0,219	0,206	0,215	0,107	0,215
clinical_ldl_c	-1,672	-0,485	-0,685	-0,714	-0,674	-0,701	-0,351	-0,702
ldl_c	0,432	0,125	0,177	0,185	0,174	0,181	0,091	0,182
hdl_c	-0,548	-0,159	-0,225	-0,234	-0,221	-0,23	-0,115	-0,23
total_tg	-0,209	-0,061	-0,086	-0,089	-0,084	-0,088	-0,044	-0,088
vldl_tg	-0,035	-0,01	-0,014	-0,015	-0,014	-0,015	-0,007	-0,015
ldl_tg	-0,062	-0,018	-0,025	-0,026	-0,025	-0,026	-0,013	-0,026
hdl_tg	-0,236	-0,068	-0,097	-0,101	-0,095	-0,099	-0,049	-0,099
total_pl	0,075	0,022	0,031	0,032	0,03	0,032	0,016	0,032
vldl_pl	1,461	0,424	0,599	0,624	0,589	0,612	0,306	0,614
ldl_pl	-0,506	-0,147	-0,207	-0,216	-0,204	-0,212	-0,106	-0,212
hdl_pl	-0,092	-0,027	-0,038	-0,039	-0,037	-0,039	-0,019	-0,039
total_ce	0,901	0,262	0,369	0,385	0,363	0,378	0,189	0,379
vldl_ce	0,136	0,04	0,056	0,058	0,055	0,057	0,029	0,057

ldl_ce	-0,401	-0,116	-0,164	-0,171	-0,162	-0,168	-0,084	-0,168
hdl_ce	-0,052	-0,015	-0,021	-0,022	-0,021	-0,022	-0,011	-0,022
total_fc	-0,83	-0,241	-0,34	-0,354	-0,334	-0,348	-0,174	-0,349
vldl_fc	0,281	0,082	0,115	0,12	0,113	0,118	0,059	0,118
ldl_fc	0	0	0	0	0	0	0	0
hdl_fc	0,007	0,002	0,003	0,003	0,003	0,003	0,002	0,003
total_l	-0,025	-0,007	-0,01	-0,011	-0,01	-0,011	-0,005	-0,011
vldl_l	-0,346	-0,1	-0,142	-0,148	-0,139	-0,145	-0,073	-0,145
ldl_l	0,211	0,061	0,086	0,09	0,085	0,088	0,044	0,088
hdl_l	0	0	0	0	0	0	0	0
total_p	-0,022	-0,006	-0,009	-0,009	-0,009	-0,009	-0,005	-0,009
vldl_p	0,366	0,106	0,15	0,156	0,148	0,153	0,077	0,154
ldl_p	0,044	0,013	0,018	0,019	0,018	0,018	0,009	0,018
hdl_p	-0,007	-0,002	-0,003	-0,003	-0,003	-0,003	-0,001	-0,003
vldl_size	-0,222	-0,064	-0,091	-0,095	-0,089	-0,093	-0,046	-0,093
ldl_size	-0,006	-0,002	-0,003	-0,003	-0,002	-0,003	-0,001	-0,003
hdl_size	-1,486	-0,431	-0,609	-0,635	-0,599	-0,623	-0,312	-0,624
phosphoglyc	0,502	0,146	0,206	0,214	0,202	0,21	0,105	0,211
tg_by_pg	-0,263	-0,076	-0,108	-0,112	-0,106	-0,11	-0,055	-0,111
cholines	-2,148	-0,623	-0,881	-0,918	-0,866	-0,9	-0,451	-0,902
phosphatidylc	0,62	0,18	0,254	0,265	0,25	0,26	0,13	0,26
sphingomyelins	0,452	0,131	0,185	0,193	0,182	0,189	0,095	0,19
apob	0,177	0,051	0,072	0,076	0,071	0,074	0,037	0,074
apoa1	0,692	0,201	0,284	0,296	0,279	0,29	0,145	0,291
apob_by_apoa1	-0,082	-0,024	-0,033	-0,035	-0,033	-0,034	-0,017	-0,034
total_fa	1,278	0,371	0,524	0,546	0,515	0,536	0,268	0,537
unsaturation	0,603	0,175	0,247	0,258	0,243	0,253	0,127	0,253
omega_3	-0,15	-0,043	-0,061	-0,064	-0,06	-0,063	-0,031	-0,063
omega_6	0,016	0,005	0,007	0,007	0,006	0,007	0,003	0,007
pufa	-0,016	-0,005	-0,007	-0,007	-0,007	-0,007	-0,003	-0,007

mufa	0,352	0,102	0,144	0,15	0,142	0,147	0,074	0,148
sfa	0,204	0,059	0,084	0,087	0,082	0,086	0,043	0,086
la	0,058	0,017	0,024	0,025	0,023	0,024	0,012	0,024
dha	0,036	0,01	0,015	0,015	0,014	0,015	0,008	0,015
omega_3_pct	-0,969	-0,281	-0,397	-0,414	-0,391	-0,406	-0,203	-0,407
omega_6_pct	-0,839	-0,243	-0,344	-0,358	-0,338	-0,352	-0,176	-0,352
pufa_pct	-0,131	-0,038	-0,054	-0,056	-0,053	-0,055	-0,028	-0,055
mufa_pct	0,334	0,097	0,137	0,143	0,135	0,14	0,07	0,14
sfa_pct	-0,079	-0,023	-0,032	-0,034	-0,032	-0,033	-0,017	-0,033
la_pct	-0,215	-0,062	-0,088	-0,092	-0,087	-0,09	-0,045	-0,09
dha_pct	-0,029	-0,008	-0,012	-0,012	-0,012	-0,012	-0,006	-0,012
pufa_by_mufa	0,861	0,25	0,353	0,368	0,347	0,361	0,181	0,361
omega_6_by_omega_3	0,032	0,009	0,013	0,014	0,013	0,014	0,007	0,014
ala	0,075	0,022	0,031	0,032	0,03	0,032	0,016	0,032
gln	0,021	0,006	0,009	0,009	0,009	0,009	0,005	0,009
gly	-0,252	-0,073	-0,103	-0,108	-0,102	-0,106	-0,053	-0,106
his	-0,086	-0,025	-0,035	-0,037	-0,035	-0,036	-0,018	-0,036
total_bcaa	0	0	0	0	0	0	0	0
ile	0,206	0,06	0,085	0,088	0,083	0,086	0,043	0,087
leu	-0,308	-0,089	-0,126	-0,132	-0,124	-0,129	-0,065	-0,129
val	-0,025	-0,007	-0,01	-0,011	-0,01	-0,011	-0,005	-0,011
phe	0,063	0,018	0,026	0,027	0,025	0,026	0,013	0,026
tyr	0,121	0,035	0,049	0,052	0,049	0,051	0,025	0,051
glucose	0,255	0,074	0,105	0,109	0,103	0,107	0,054	0,107
lactate	0,168	0,049	0,069	0,072	0,068	0,071	0,035	0,071
pyruvate	-0,096	-0,028	-0,039	-0,041	-0,039	-0,04	-0,02	-0,04
citrate	-0,081	-0,023	-0,033	-0,034	-0,032	-0,034	-0,017	-0,034
acetate	0,02	0,006	0,008	0,009	0,008	0,009	0,004	0,009
acetoacetate	-0,022	-0,006	-0,009	-0,009	-0,009	-0,009	-0,005	-0,009
acetone	0,037	0,011	0,015	0,016	0,015	0,016	0,008	0,016

albumin	-0,004	-0,001	-0,002	-0,002	-0,002	-0,002	-0,001	-0,002
glyca	0,064	0,018	0,026	0,027	0,026	0,027	0,013	0,027
xxl_vldl_p	-1,256	-0,364	-0,515	-0,537	-0,506	-0,527	-0,263	-0,528
xxl_vldl_l	-0,671	-0,195	-0,275	-0,286	-0,27	-0,281	-0,141	-0,282
xxl_vldl_pl	1,288	0,374	0,528	0,55	0,519	0,54	0,27	0,541
xxl_vldl_c	0,001	0	0	0	0	0	0	0
xxl_vldl_ce	0,052	0,015	0,021	0,022	0,021	0,022	0,011	0,022
xxl_vldl_fc	-0,131	-0,038	-0,054	-0,056	-0,053	-0,055	-0,027	-0,055
xxl_vldl_tg	0,079	0,023	0,032	0,034	0,032	0,033	0,016	0,033
xl_vldl_p	-1,263	-0,366	-0,518	-0,539	-0,509	-0,529	-0,265	-0,53
xl_vldl_l	-0,008	-0,002	-0,003	-0,003	-0,003	-0,003	-0,002	-0,003
xl_vldl_pl	0,247	0,072	0,101	0,106	0,1	0,104	0,052	0,104
xl_vldl_c	0,099	0,029	0,041	0,042	0,04	0,042	0,021	0,042
xl_vldl_ce	-0,17	-0,049	-0,07	-0,073	-0,069	-0,071	-0,036	-0,071
xl_vldl_fc	0,159	0,046	0,065	0,068	0,064	0,067	0,033	0,067
xl_vldl_tg	-0,416	-0,121	-0,171	-0,178	-0,168	-0,175	-0,087	-0,175
l_vldl_p	-0,156	-0,045	-0,064	-0,067	-0,063	-0,065	-0,033	-0,066
l_vldl_l	0,372	0,108	0,153	0,159	0,15	0,156	0,078	0,156
l_vldl_pl	0,025	0,007	0,01	0,011	0,01	0,01	0,005	0,01
l_vldl_c	-0,638	-0,185	-0,261	-0,272	-0,257	-0,267	-0,134	-0,268
l_vldl_ce	-0,002	-0,001	-0,001	-0,001	-0,001	-0,001	0	-0,001
l_vldl_fc	-0,268	-0,078	-0,11	-0,115	-0,108	-0,112	-0,056	-0,113
l_vldl_tg	1,301	0,378	0,533	0,556	0,524	0,545	0,273	0,547
m_vldl_p	0,148	0,043	0,061	0,063	0,06	0,062	0,031	0,062
m_vldl_l	-0,4	-0,116	-0,164	-0,171	-0,161	-0,168	-0,084	-0,168
m_vldl_pl	-0,168	-0,049	-0,069	-0,072	-0,068	-0,07	-0,035	-0,071
m_vldl_c	-0,159	-0,046	-0,065	-0,068	-0,064	-0,067	-0,033	-0,067
m_vldl_ce	-0,201	-0,058	-0,082	-0,086	-0,081	-0,084	-0,042	-0,084
m_vldl_fc	0,324	0,094	0,133	0,138	0,13	0,136	0,068	0,136
m_vldl_tg	0,018	0,005	0,007	0,008	0,007	0,007	0,004	0,007

s_vldl_p	0,282	0,082	0,115	0,12	0,114	0,118	0,059	0,118
s_vldl_l	-0,044	-0,013	-0,018	-0,019	-0,018	-0,019	-0,009	-0,019
s_vldl_pl	1,539	0,447	0,631	0,657	0,62	0,645	0,323	0,646
s_vldl_c	-0,418	-0,121	-0,171	-0,179	-0,169	-0,175	-0,088	-0,176
s_vldl_ce	-0,061	-0,018	-0,025	-0,026	-0,025	-0,025	-0,013	-0,026
s_vldl_fc	-0,205	-0,06	-0,084	-0,088	-0,083	-0,086	-0,043	-0,086
s_vldl_tg	-1,459	-0,423	-0,598	-0,623	-0,588	-0,612	-0,306	-0,613
xs_vldl_p	-0,583	-0,169	-0,239	-0,249	-0,235	-0,244	-0,122	-0,245
xs_vldl_l	-0,006	-0,002	-0,002	-0,003	-0,002	-0,003	-0,001	-0,003
xs_vldl_pl	0,815	0,236	0,334	0,348	0,328	0,341	0,171	0,342
xs_vldl_c	-0,955	-0,277	-0,392	-0,408	-0,385	-0,4	-0,2	-0,401
xs_vldl_ce	-0,232	-0,067	-0,095	-0,099	-0,093	-0,097	-0,049	-0,097
xs_vldl_fc	0,255	0,074	0,105	0,109	0,103	0,107	0,054	0,107
xs_vldl_tg	-0,444	-0,129	-0,182	-0,19	-0,179	-0,186	-0,093	-0,186
idl_p	-0,784	-0,227	-0,321	-0,335	-0,316	-0,329	-0,164	-0,329
idl_l	0,629	0,182	0,258	0,268	0,253	0,263	0,132	0,264
idl_pl	-0,762	-0,221	-0,312	-0,325	-0,307	-0,319	-0,16	-0,32
idl_c	0,847	0,246	0,347	0,362	0,341	0,355	0,178	0,356
idl_ce	-0,417	-0,121	-0,171	-0,178	-0,168	-0,175	-0,087	-0,175
idl_fc	2,768	0,803	1,134	1,182	1,115	1,16	0,581	1,162
idl_tg	0,544	0,158	0,223	0,232	0,219	0,228	0,114	0,228
l_ldl_p	-1,592	-0,462	-0,653	-0,68	-0,642	-0,667	-0,334	-0,669
l_ldl_l	-0,392	-0,114	-0,161	-0,167	-0,158	-0,164	-0,082	-0,165
l_ldl_pl	-1,071	-0,311	-0,439	-0,457	-0,432	-0,449	-0,225	-0,45
l_ldl_c	1,017	0,295	0,417	0,434	0,41	0,426	0,213	0,427
l_ldl_ce	0,367	0,107	0,151	0,157	0,148	0,154	0,077	0,154
l_ldl_fc	-1,134	-0,329	-0,465	-0,484	-0,457	-0,475	-0,238	-0,476
l_ldl_tg	0,28	0,081	0,115	0,119	0,113	0,117	0,059	0,117
m_ldl_p	-0,367	-0,107	-0,151	-0,157	-0,148	-0,154	-0,077	-0,154
m_ldl_l	-0,6	-0,174	-0,246	-0,256	-0,242	-0,252	-0,126	-0,252

m_ldl_pl	0,465	0,135	0,19	0,198	0,187	0,195	0,097	0,195
m_ldl_c	-0,71	-0,206	-0,291	-0,303	-0,286	-0,297	-0,149	-0,298
m_ldl_ce	-0,309	-0,09	-0,127	-0,132	-0,125	-0,13	-0,065	-0,13
m_ldl_fc	0,633	0,184	0,259	0,27	0,255	0,265	0,133	0,266
m_ldl_tg	-0,022	-0,006	-0,009	-0,009	-0,009	-0,009	-0,005	-0,009
s_ldl_p	0,019	0,006	0,008	0,008	0,008	0,008	0,004	0,008
s_ldl_l	-0,068	-0,02	-0,028	-0,029	-0,027	-0,028	-0,014	-0,028
s_ldl_pl	-0,236	-0,069	-0,097	-0,101	-0,095	-0,099	-0,05	-0,099
s_ldl_c	0,092	0,027	0,038	0,039	0,037	0,039	0,019	0,039
s_ldl_ce	0	0	0	0	0	0	0	0
s_ldl_fc	0,167	0,048	0,068	0,071	0,067	0,07	0,035	0,07
s_ldl_tg	0,22	0,064	0,09	0,094	0,089	0,092	0,046	0,093
xl_hdl_p	-0,964	-0,28	-0,395	-0,412	-0,389	-0,404	-0,202	-0,405
xl_hdl_l	-0,555	-0,161	-0,227	-0,237	-0,224	-0,232	-0,116	-0,233
xl_hdl_pl	-0,637	-0,185	-0,261	-0,272	-0,257	-0,267	-0,134	-0,268
xl_hdl_c	0,771	0,224	0,316	0,329	0,311	0,323	0,162	0,324
xl_hdl_ce	0	0	0	0	0	0	0	0
xl_hdl_fc	0,522	0,151	0,214	0,223	0,21	0,219	0,109	0,219
xl_hdl_tg	0,658	0,191	0,27	0,281	0,265	0,276	0,138	0,276
l_hdl_p	-0,03	-0,009	-0,012	-0,013	-0,012	-0,013	-0,006	-0,013
l_hdl_l	-0,287	-0,083	-0,118	-0,123	-0,116	-0,12	-0,06	-0,121
l_hdl_pl	-0,102	-0,03	-0,042	-0,043	-0,041	-0,043	-0,021	-0,043
l_hdl_c	0,153	0,045	0,063	0,066	0,062	0,064	0,032	0,064
l_hdl_ce	0,061	0,018	0,025	0,026	0,025	0,026	0,013	0,026
l_hdl_fc	0,074	0,021	0,03	0,032	0,03	0,031	0,016	0,031
l_hdl_tg	-0,316	-0,092	-0,13	-0,135	-0,127	-0,133	-0,066	-0,133
m_hdl_p	-0,347	-0,101	-0,142	-0,148	-0,14	-0,146	-0,073	-0,146
m_hdl_l	-0,223	-0,065	-0,092	-0,095	-0,09	-0,094	-0,047	-0,094
m_hdl_pl	-0,263	-0,076	-0,108	-0,112	-0,106	-0,11	-0,055	-0,11
m_hdl_c	0,186	0,054	0,076	0,08	0,075	0,078	0,039	0,078

m_hdl_ce	0,038	0,011	0,016	0,016	0,015	0,016	0,008	0,016
m_hdl_fc	0,52	0,151	0,213	0,222	0,21	0,218	0,109	0,219
m_hdl_tg	-0,484	-0,14	-0,198	-0,207	-0,195	-0,203	-0,102	-0,203
s_hdl_p	-0,224	-0,065	-0,092	-0,095	-0,09	-0,094	-0,047	-0,094
s_hdl_l	0,007	0,002	0,003	0,003	0,003	0,003	0,001	0,003
s_hdl_pl	-0,052	-0,015	-0,021	-0,022	-0,021	-0,022	-0,011	-0,022
s_hdl_c	-0,009	-0,003	-0,004	-0,004	-0,004	-0,004	-0,002	-0,004
s_hdl_ce	0,019	0,005	0,008	0,008	0,008	0,008	0,004	0,008
s_hdl_fc	-0,043	-0,013	-0,018	-0,019	-0,018	-0,018	-0,009	-0,018
s_hdl_tg	0,017	0,005	0,007	0,007	0,007	0,007	0,004	0,007
xl_vldl_pl_pct	0,013	0,004	0,006	0,006	0,005	0,006	0,003	0,006
xl_vldl_c_pct	0,024	0,007	0,01	0,01	0,01	0,01	0,005	0,01
xl_vldl_ce_pct	0	0	0	0	0	0	0	0
xl_vldl_fc_pct	-0,04	-0,012	-0,017	-0,017	-0,016	-0,017	-0,008	-0,017
xl_vldl_tg_pct	0,017	0,005	0,007	0,007	0,007	0,007	0,004	0,007
l_vldl_pl_pct	-0,064	-0,019	-0,026	-0,027	-0,026	-0,027	-0,013	-0,027
l_vldl_c_pct	-0,104	-0,03	-0,043	-0,044	-0,042	-0,044	-0,022	-0,044
l_vldl_ce_pct	-0,19	-0,055	-0,078	-0,081	-0,076	-0,079	-0,04	-0,08
l_vldl_fc_pct	0,317	0,092	0,13	0,136	0,128	0,133	0,067	0,133
l_vldl_tg_pct	0,12	0,035	0,049	0,051	0,048	0,05	0,025	0,05
m_vldl_pl_pct	0,285	0,083	0,117	0,122	0,115	0,119	0,06	0,12
m_vldl_c_pct	0,239	0,069	0,098	0,102	0,097	0,1	0,05	0,101
m_vldl_ce_pct	0,056	0,016	0,023	0,024	0,023	0,023	0,012	0,024
m_vldl_fc_pct	-0,44	-0,128	-0,18	-0,188	-0,177	-0,184	-0,092	-0,185
m_vldl_tg_pct	-0,309	-0,09	-0,127	-0,132	-0,125	-0,13	-0,065	-0,13
s_vldl_pl_pct	0,147	0,043	0,06	0,063	0,059	0,062	0,031	0,062
s_vldl_c_pct	-0,316	-0,092	-0,129	-0,135	-0,127	-0,132	-0,066	-0,133
s_vldl_ce_pct	-0,041	-0,012	-0,017	-0,017	-0,016	-0,017	-0,009	-0,017
s_vldl_fc_pct	-0,223	-0,065	-0,091	-0,095	-0,09	-0,093	-0,047	-0,094
s_vldl_tg_pct	0,128	0,037	0,052	0,055	0,052	0,054	0,027	0,054

xs_vldl_pl_pct	-0,246	-0,071	-0,101	-0,105	-0,099	-0,103	-0,052	-0,103
xs_vldl_c_pct	-0,339	-0,098	-0,139	-0,145	-0,137	-0,142	-0,071	-0,142
xs_vldl_ce_pct	-0,036	-0,011	-0,015	-0,015	-0,015	-0,015	-0,008	-0,015
xs_vldl_fc_pct	0,349	0,101	0,143	0,149	0,141	0,146	0,073	0,147
xs_vldl_tg_pct	0,308	0,089	0,126	0,132	0,124	0,129	0,065	0,129
idl_pl_pct	0,625	0,181	0,256	0,267	0,252	0,262	0,131	0,263
idl_c_pct	0,06	0,017	0,025	0,026	0,024	0,025	0,013	0,025
idl_ce_pct	0,199	0,058	0,082	0,085	0,08	0,084	0,042	0,084
idl_fc_pct	-0,54	-0,157	-0,221	-0,231	-0,218	-0,226	-0,113	-0,227
idl_tg_pct	-0,196	-0,057	-0,081	-0,084	-0,079	-0,082	-0,041	-0,082
l_ldl_pl_pct	0,09	0,026	0,037	0,038	0,036	0,038	0,019	0,038
l_ldl_c_pct	-0,128	-0,037	-0,053	-0,055	-0,052	-0,054	-0,027	-0,054
l_ldl_ce_pct	0,003	0,001	0,001	0,001	0,001	0,001	0,001	0,001
l_ldl_fc_pct	0,088	0,025	0,036	0,037	0,035	0,037	0,018	0,037
l_ldl_tg_pct	-0,024	-0,007	-0,01	-0,01	-0,01	-0,01	-0,005	-0,01
m_ldl_pl_pct	-0,025	-0,007	-0,01	-0,011	-0,01	-0,01	-0,005	-0,01
m_ldl_c_pct	0,301	0,087	0,123	0,128	0,121	0,126	0,063	0,126
m_ldl_ce_pct	-0,15	-0,043	-0,061	-0,064	-0,06	-0,063	-0,031	-0,063
m_ldl_fc_pct	-0,138	-0,04	-0,057	-0,059	-0,056	-0,058	-0,029	-0,058
m_ldl_tg_pct	-0,008	-0,002	-0,003	-0,004	-0,003	-0,003	-0,002	-0,004
s_ldl_pl_pct	-0,06	-0,017	-0,024	-0,025	-0,024	-0,025	-0,013	-0,025
s_ldl_c_pct	0,102	0,03	0,042	0,044	0,041	0,043	0,021	0,043
s_ldl_ce_pct	-0,204	-0,059	-0,084	-0,087	-0,082	-0,085	-0,043	-0,086
s_ldl_fc_pct	0,094	0,027	0,039	0,04	0,038	0,04	0,02	0,04
s_ldl_tg_pct	0,17	0,049	0,07	0,073	0,069	0,071	0,036	0,072
xl_hdl_pl_pct	-0,038	-0,011	-0,016	-0,016	-0,015	-0,016	-0,008	-0,016
xl_hdl_c_pct	0,001	0	0	0	0	0	0	0
xl_hdl_ce_pct	0,03	0,009	0,012	0,013	0,012	0,013	0,006	0,013
xl_hdl_fc_pct	-0,033	-0,01	-0,014	-0,014	-0,013	-0,014	-0,007	-0,014
xl_hdl_tg_pct	0,012	0,004	0,005	0,005	0,005	0,005	0,003	0,005

l_hdl_pl_pct	-0,04	-0,012	-0,016	-0,017	-0,016	-0,017	-0,008	-0,017
l_hdl_c_pct	0,059	0,017	0,024	0,025	0,024	0,025	0,012	0,025
l_hdl_ce_pct	0	0	0	0	0	0	0	0
l_hdl_fc_pct	0,048	0,014	0,02	0,02	0,019	0,02	0,01	0,02
l_hdl_tg_pct	0,008	0,002	0,003	0,003	0,003	0,003	0,002	0,003
m_hdl_pl_pct	-0,373	-0,108	-0,153	-0,159	-0,15	-0,156	-0,078	-0,157
m_hdl_c_pct	-0,474	-0,138	-0,194	-0,203	-0,191	-0,199	-0,099	-0,199
m_hdl_ce_pct	0,305	0,088	0,125	0,13	0,123	0,128	0,064	0,128
m_hdl_fc_pct	-0,052	-0,015	-0,021	-0,022	-0,021	-0,022	-0,011	-0,022
m_hdl_tg_pct	-0,127	-0,037	-0,052	-0,054	-0,051	-0,053	-0,027	-0,053
s_hdl_pl_pct	0,456	0,132	0,187	0,195	0,184	0,191	0,096	0,192
s_hdl_c_pct	-0,002	-0,001	-0,001	-0,001	-0,001	-0,001	0	-0,001
s_hdl_ce_pct	-0,072	-0,021	-0,029	-0,031	-0,029	-0,03	-0,015	-0,03
s_hdl_fc_pct	0,551	0,16	0,226	0,235	0,222	0,231	0,116	0,231
s_hdl_tg_pct	0,002	0,001	0,001	0,001	0,001	0,001	0	0,001
