# Supplementary material for: Penalized Reduced Rank Regression for Multi‐Outcome Survival Data Supports a Common Metabolic Risk Score for Age‐Related Diseases
Source: Stat Med. 2025 Jul 15;44(15-17):e70156. doi: 10.1002/sim.70156 (PMC12261392; doi:10.1002/sim.70156)
Supplement: Supplementary file 8 — Data S8. Supporting Information S8. [file SIM-44-0-s009.pdf]

Supporting Information to "Penalized reduced rank regression for multi-outcome survival data supports a common metabolic risk score for age-related diseases"

Marije H. Sluiskes[1], Hein Putter[1], Marian Beekman[1],
Jelle J. Goeman[1] and Mar Rodríguez-Girondo[1]

[1]Biomedical Data Sciences, Leiden University Medical Center,
Einthovenweg 20, 2333 ZC Leiden, The Netherlands

SUPPORTING TABLE 6 Estimated coefficient matrix $\hat{\boldsymbol{A}}$ of optimal model.

metabolic_variable	coefficient
total_c	0,253
non_hdl_c	0,040
remnant_c	0,173
vldl_c	0,140
clinical_ldl_c	-0,459
ldl_c	0,119
hdl_c	-0,150
total_tg	-0,057
vldl_tg	-0,010
ldl_tg	-0,017
hdl_tg	-0,065
total_pl	0,021
vldl_pl	0,401
ldl_pl	-0,139
hdl_pl	-0,025
total_ce	0,247
vldl_ce	0,037
ldl_ce	-0,110
hdl_ce	-0,014
total_fc	-0,228
vldl_fc	0,077
ldl_fc	0,000
hdl_fc	0,002
total_l	-0,007
vldl_l	-0,095
ldl_l	0,058
hdl_l	0,000
total_p	-0,006
vldl_p	0,100
ldl_p	0,012
hdl_p	-0,002
vldl_size	-0,061

ldl_size	-0,002
hdl_size	-0,408
phosphoglyc	0,138
tg_by_pg	-0,072
cholines	-0,589
phosphatidylc	0,170
sphingomyelins	0,124
apob	0,049
apoa1	0,190
apob_by_apoa1	-0,022
total_fa	0,351
unsaturation	0,166
omega_3	-0,041
omega_6	0,004
pufa	-0,004
mufa	0,096
sfa	0,056
la	0,016
dha	0,010
omega_3_pct	-0,266
omega_6_pct	-0,230
pufa_pct	-0,036
mufa_pct	0,092
sfa_pct	-0,022
la_pct	-0,059
dha_pct	-0,008
pufa_by_mufa	0,236
omega_6_by_omega_3	0,009
ala	0,021
gln	0,006
gly	-0,069
his	-0,024
total_bcaa	0,000
ile	0,057
leu	-0,084
val	-0,007
phe	0,017
tyr	0,033
glucose	0,070
lactate	0,046
pyruvate	-0,026
citrate	-0,022
acetate	0,006
acetoacetate	-0,006
acetone	0,010
albumin	-0,001

glyca	0,017
xxl_vldl_p	-0,345
xxl_vldl_l	-0,184
xxl_vldl_pl	0,354
xxl_vldl_c	0,000
xxl_vldl_ce	0,014
xxl_vldl_fc	-0,036
xxl_vldl_tg	0,022
xl_vldl_p	-0,347
xl_vldl_l	-0,002
xl_vldl_pl	0,068
xl_vldl_c	0,027
xl_vldl_ce	-0,047
xl_vldl_fc	0,044
xl_vldl_tg	-0,114
l_vldl_p	-0,043
l_vldl_l	0,102
l_vldl_pl	0,007
l_vldl_c	-0,175
l_vldl_ce	-0,001
l_vldl_fc	-0,074
l_vldl_tg	0,357
m_vldl_p	0,041
m_vldl_l	-0,110
m_vldl_pl	-0,046
m_vldl_c	-0,044
m_vldl_ce	-0,055
m_vldl_fc	0,089
m_vldl_tg	0,005
s_vldl_p	0,077
s_vldl_l	-0,012
s_vldl_pl	0,422
s_vldl_c	-0,115
s_vldl_ce	-0,017
s_vldl_fc	-0,056
s_vldl_tg	-0,400
xs_vldl_p	-0,160
xs_vldl_l	-0,002
xs_vldl_pl	0,224
xs_vldl_c	-0,262
xs_vldl_ce	-0,064
xs_vldl_fc	0,070
xs_vldl_tg	-0,122
idl_p	-0,215
idl_l	0,172
idl_pl	-0,209

idl_c	0,232
idl_ce	-0,114
idl_fc	0,759
idl_tg	0,149
l_ldl_p	-0,437
l_ldl_l	-0,108
l_ldl_pl	-0,294
l_ldl_c	0,279
l_ldl_ce	0,101
l_ldl_fc	-0,311
l_ldl_tg	0,077
m_ldl_p	-0,101
m_ldl_l	-0,165
m_ldl_pl	0,128
m_ldl_c	-0,195
m_ldl_ce	-0,085
m_ldl_fc	0,174
m_ldl_tg	-0,006
s_ldl_p	0,005
s_ldl_l	-0,019
s_ldl_pl	-0,065
s_ldl_c	0,025
s_ldl_ce	0,000
s_ldl_fc	0,046
s_ldl_tg	0,060
xl_hdl_p	-0,265
xl_hdl_l	-0,152
xl_hdl_pl	-0,175
xl_hdl_c	0,212
xl_hdl_ce	0,000
xl_hdl_fc	0,143
xl_hdl_tg	0,181
l_hdl_p	-0,008
l_hdl_l	-0,079
l_hdl_pl	-0,028
l_hdl_c	0,042
l_hdl_ce	0,017
l_hdl_fc	0,020
l_hdl_tg	-0,087
m_hdl_p	-0,095
m_hdl_l	-0,061
m_hdl_pl	-0,072
m_hdl_c	0,051
m_hdl_ce	0,010
m_hdl_fc	0,143
m_hdl_tg	-0,133

s_hdl_p	-0,061
s_hdl_l	0,002
s_hdl_pl	-0,014
s_hdl_c	-0,002
s_hdl_ce	0,005
s_hdl_fc	-0,012
s_hdl_tg	0,005
xl_vldl_pl_pct	0,004
xl_vldl_c_pct	0,007
xl_vldl_ce_pct	0,000
xl_vldl_fc_pct	-0,011
xl_vldl_tg_pct	0,005
l_vldl_pl_pct	-0,018
l_vldl_c_pct	-0,029
l_vldl_ce_pct	-0,052
l_vldl_fc_pct	0,087
l_vldl_tg_pct	0,033
m_vldl_pl_pct	0,078
m_vldl_c_pct	0,066
m_vldl_ce_pct	0,015
m_vldl_fc_pct	-0,121
m_vldl_tg_pct	-0,085
s_vldl_pl_pct	0,040
s_vldl_c_pct	-0,087
s_vldl_ce_pct	-0,011
s_vldl_fc_pct	-0,061
s_vldl_tg_pct	0,035
xs_vldl_pl_pct	-0,067
xs_vldl_c_pct	-0,093
xs_vldl_ce_pct	-0,010
xs_vldl_fc_pct	0,096
xs_vldl_tg_pct	0,085
idl_pl_pct	0,172
idl_c_pct	0,017
idl_ce_pct	0,055
idl_fc_pct	-0,148
idl_tg_pct	-0,054
l_ldl_pl_pct	0,025
l_ldl_c_pct	-0,035
l_ldl_ce_pct	0,001
l_ldl_fc_pct	0,024
l_ldl_tg_pct	-0,006
m_ldl_pl_pct	-0,007
m_ldl_c_pct	0,083
m_ldl_ce_pct	-0,041
m_ldl_fc_pct	-0,038

m_ldl_tg_pct	-0,002
s_ldl_pl_pct	-0,016
s_ldl_c_pct	0,028
s_ldl_ce_pct	-0,056
s_ldl_fc_pct	0,026
s_ldl_tg_pct	0,047
xl_hdl_pl_pct	-0,010
xl_hdl_c_pct	0,000
xl_hdl_ce_pct	0,008
xl_hdl_fc_pct	-0,009
xl_hdl_tg_pct	0,003
l_hdl_pl_pct	-0,011
l_hdl_c_pct	0,016
l_hdl_ce_pct	0,000
l_hdl_fc_pct	0,013
l_hdl_tg_pct	0,002
m_hdl_pl_pct	-0,102
m_hdl_c_pct	-0,130
m_hdl_ce_pct	0,084
m_hdl_fc_pct	-0,014
m_hdl_tg_pct	-0,035
s_hdl_pl_pct	0,125
s_hdl_c_pct	-0,001
s_hdl_ce_pct	-0,020
s_hdl_fc_pct	0,151
s_hdl_tg_pct	0,001
